# The Complexity of Managing Antipsychotic-Induced Hyponatremia: A Case Series

**DOI:** 10.7759/cureus.71038

**Published:** 2024-10-07

**Authors:** Syed Ali Bokhari, Dalal Al-Maktoum, Ghina Alramahi, Daliya Maallah, Mazin A Mukhtar

**Affiliations:** 1 Psychiatry, Al Amal Psychiatric Hospital, Emirates Health Services, Dubai, ARE

**Keywords:** antipsychotic-induced hyponatremia, antipsychotics, electrolyte imbalance, hyponatremia, lithium, psychotropic-induced hyponatremia, serum sodium levels, sodium imbalance, syndrome of inappropriate secretion of antidiuretic hormone (siadh), water-electrolyte balance

## Abstract

This case series examines the development of hyponatremia in three patients following the administration of antipsychotic medications. Hyponatremia, a common and potentially serious electrolyte imbalance, can complicate the treatment of psychiatric disorders, particularly when antipsychotic medications are involved. The cases presented demonstrate the fluctuating sodium levels in patients and the persistent challenge of managing both psychiatric stability and electrolyte balance. In all three cases, sodium levels were closely monitored and required frequent interventions to prevent further decline. Regular monitoring and targeted adjustments, such as the introduction of lithium for its mood-stabilizing effects and sodium-balancing properties and aripiprazole for its lower risk of causing syndrome of inappropriate antidiuretic hormone secretion, played a critical role in stabilizing both sodium levels and psychiatric symptoms. The clinical complexity of these cases highlights the critical need for a tailored, evolving approach in managing antipsychotic-induced hyponatremia. Effective management requires precise attention to fluid and sodium regulation while maintaining psychiatric stabilization and preventing relapse. This case series underscores the importance of continuous sodium monitoring and timely medication adjustments to ensure long-term psychiatric and medical stability. The dynamic interplay between psychiatric symptoms and electrolyte disturbances demands a personalized, interdisciplinary strategy to achieve therapeutic success in such challenging and multifaceted cases.

## Introduction

Hyponatremia, defined as a serum sodium concentration below 135 mmol/L, is a frequent and potentially serious electrolyte disorder observed in hospitalized patients. It is associated with increased morbidity and mortality, particularly when it develops acutely or when serum sodium levels drop to severely low levels. The condition occurs due to an imbalance between water and sodium in the body, often leading to an excess of water relative to sodium in the extracellular fluid, which results in a dilutional decrease in sodium concentration [1].

In psychiatric practice, the use of antipsychotic medications, particularly second-generation antipsychotics (SGAs), has raised concerns about the risk of developing hyponatremia. Several studies have implicated antipsychotics in the onset of hyponatremia, primarily through mechanisms involving SIADH or nephrogenic syndrome of inappropriate antidiuresis (NSIAD). These mechanisms involve the stimulation of the vasopressin V2 receptor (V2R) in the kidneys, leading to increased water reabsorption independent of antidiuretic hormone (ADH) levels, which contributes to dilutional hyponatremia. Despite the critical role antipsychotic medications play in managing psychiatric disorders such as schizophrenia, their association with hyponatremia presents a significant clinical challenge. The risk of hyponatremia may be exacerbated by the syndrome of inappropriate antidiuretic hormone secretion (SIADH), which leads to water retention and dilutional hyponatremia [2].

This case report aims to explore the correlation between antipsychotic use and the development of hyponatremia by examining three cases in which patients developed electrolyte disturbance following the initiation or continuation of antipsychotic therapy. Additionally, it highlights the treatment dilemma clinicians face when hyponatremia complicates psychiatric management, often forcing difficult decisions about altering or discontinuing antipsychotic medications, thereby risking psychiatric destabilization. The findings from these cases are discussed in the context of existing literature, underscoring the need for heightened clinical awareness and individualized treatment strategies.

## Case presentation

Case 1

A 49-year-old South Asian male with a 20-year history of schizophrenia, married with seven children, was admitted to a tertiary psychiatric facility following a series of aggressive outbursts, auditory hallucinations, and nonadherence to his medication regimen. His psychiatric symptoms had been poorly controlled over the years, with multiple hospitalizations due to noncompliance with treatment. He was managed initially on oral olanzapine 20 mg daily and long-acting haloperidol decanoate injections at 100 mg intramuscularly every four weeks. He was also later started on quetiapine 300 mg, eventually titrated up to 300 mg twice daily. Sodium levels at admission were within the normal range, but within a month of starting olanzapine, his sodium levels dropped to 133 mmol/L. No immediate corrective action was taken as the decline was mild, but his psychiatric symptoms, particularly his agitation and hallucinations, worsened.

Over the following year, the patient continued to experience worsening psychosis and was readmitted with severe agitation, increased aggression, and disorganized behavior. During this admission, he was initiated on sodium valproate 500 mg twice daily. His sodium levels dropped further to 126 mmol/L. He was treated with intravenous sodium chloride and dietary modifications aimed at correcting the hyponatremia. Oral haloperidol 5 mg twice daily was introduced alongside his existing regimen in an attempt to stabilize his psychiatric symptoms. However, despite these interventions, his sodium levels continued to decline, with a nadir of 120 mmol/L, indicating severe hyponatremia. Further evaluation revealed that sodium valproate, now titrated up to 500 mg in the morning and 1000 mg at night, was likely exacerbating the hyponatremia as well, on top of the olanzapine and haloperidol.

Approximately six months later, due to persistent hyponatremia and ongoing psychotic symptoms, a major revision of the patient’s antipsychotic regimen was undertaken. Aripiprazole was introduced as a long-acting injection at 400 mg intramuscularly monthly, while quetiapine was continued at 400 mg twice daily, and oral olanzapine and haloperidol were gradually tapered off. Following these changes, the patient’s sodium levels initially showed a transient improvement, rising to 128 mmol/L, indicating a partial response to the modification in his treatment regimen. However, despite this initial improvement, the patient experienced another relapse of psychotic symptoms, characterized by increased agitation, delusions, and auditory hallucinations, leading to further hospitalizations in the following year. During this period, sodium levels continued to fluctuate, reaching critically low values of 118 mmol/L. Repeated corrective measures were employed, including hypertonic saline infusions and stringent monitoring of sodium levels.

In response to the persistent hyponatremia and ongoing psychiatric instability, sodium valproate was gradually tapered and discontinued. Lithium 300 mg twice daily was introduced and titrated up to 1000 mg daily, to achieve therapeutic serum levels. Following this adjustment, the patient’s sodium levels gradually normalized, reaching near-normal levels of approximately 135 mmol/L. The introduction of lithium, known for its stabilizing effects on mood, psychosis, and sodium balance, significantly improved his overall clinical condition.

The combination of lithium, quetiapine, continued monthly aripiprazole injections, and dietary modifications resulted in sustained improvements in both sodium balance and psychiatric control. The patient demonstrated a marked reduction in the frequency of relapses and a significant enhancement in overall stability. This case highlights the critical challenges of managing hyponatremia caused by psychotropic medications, particularly antipsychotics, in patients with schizophrenia. This case not only emphasizes the potential role of lithium in mitigating this adverse effect but also underscores the need for a highly individualized approach to treatment, with dynamic adjustments tailored to the patient's evolving clinical needs to effectively manage both psychiatric symptoms and complex electrolyte disturbances.

Case 2

A 52-year-old Middle Eastern male, retired and living with his sibling, has a long-standing psychiatric history of schizophrenia spanning over two decades, with multiple psychiatric admissions. Despite a history of good medication compliance, he had demonstrated limited improvement between admissions. He initially received care at a general medical hospital with psychiatric services and, as of three years now, transitioned to regular follow-up at a tertiary psychiatric hospital.

The patient was first diagnosed with schizophrenia at age 20 and has undergone extensive trials of psychotropic medications. Since 2021, his treatment regimen has remained consistent, comprising aripiprazole 20 mg daily, clozapine 350 mg daily, sodium valproate 500 mg twice daily, and sertraline 150 mg daily. Despite these medications, his condition remained unstable with only mild improvements in his symptoms over several discharges, such as reduction in aggression and improved sleep. Additionally, the patient has experienced recurrent mild hyponatremia during this period, with serum sodium levels fluctuating between 127 and 134 mmol/L, first noted in 2021 and persisting into 2024.

The patient’s developmental history is unremarkable, and he has no notable medical or surgical history. There is also no family history of mental illness.

During his most recent admission, the patient presented with worsening psychotic symptoms, including increased suspiciousness, as he expressed heightened paranoia and a belief that people were constantly watching him. Additionally, he displayed an irritable mood; his brother noted that the patient's increased irritability was driven by delusional thoughts of persecution. Additionally, there were documented episodes of aggression, as reported by the patient’s brother, who described instances of physical violence, including the destruction of household items. These aggressive outbursts appeared to be precipitated by the patient’s persecutory delusions, particularly the belief that he was being followed or surveilled, further complicating the clinical picture and highlighting the severity of his psychotic symptoms. 

Over time, however, his sodium levels fluctuated, with periodic drops to 125 mmol/L during episodes of increased psychiatric distress. The decision was made to taper sodium valproate gradually and replace it with lithium, starting at 300 mg twice daily. This transition resulted in a partial improvement in sodium levels, with stabilization at around 134 mmol/L, though mild hyponatremia persisted. However, his psychiatric symptoms improved, and the frequency of hospitalizations decreased. The patient was discharged with a regular outpatient follow-up appointment; his sodium levels continued to be monitored in the clinic.

This case highlights the challenges of managing mild, recurrent hyponatremia in patients with schizophrenia who require long-term antipsychotic therapy. Lithium provided a partial solution by stabilizing both psychiatric symptoms and sodium balance, though continued monitoring was necessary to prevent further episodes of hyponatremia.

Case 3

A 62-year-old Middle Eastern male with a 30-year history of schizoaffective disorder, along with chronic kidney disease (CKD), diabetes mellitus, and dyslipidemia, presented with a history of multiple inpatient psychiatric admissions. His initial diagnosis of schizoaffective disorder in his 30s led to frequent psychotic episodes requiring repeated hospitalizations. Over the years, the patient had been treated with various psychotropic medications, including risperidone 2 mg twice daily, escitalopram 10 mg daily, and monthly paliperidone 150 mg intramuscular long-acting injections. However, poor adherence to medication significantly hindered the effectiveness of his treatment, leading to frequent relapses of his psychiatric symptoms and complicating the management of his comorbid medical conditions.

In 2022, the patient was admitted to the emergency department with severe psychotic symptoms, including agitation, paranoia, and delusions that his food was poisoned and that his family members were stealing from him. During admission, his serum sodium level was found to be critically low at 124 mmol/L, a significant decrease that prompted the medical team to investigate possible causes of hyponatremia. This condition was initially attributed to a combination of factors, including his medication regimen, renal function, and dietary habits. His CKD, which impaired the kidneys’ ability to handle fluid and sodium balance, was identified as a contributing factor. Additionally, his use of paliperidone long-acting injection, known to be associated with SIADH secretion, further exacerbated the hyponatremia. Despite this, paliperidone was continued at the time due to the lack of alternatives and the patient’s psychiatric instability.

Throughout the following months, the patient’s sodium levels fluctuated between 120 and 130 mmol/L, which necessitated frequent monitoring. His sodium levels remained low despite adjustments to his fluid intake and dietary sodium. His psychotic symptoms persisted, marked by aggressive outbursts and hallucinations, further complicating his clinical management. In early 2023, his sodium level dropped further to 118 mmol/L during another episode of psychotic relapse, characterized by auditory hallucinations and paranoia. At this point, the decision to use paliperidone was carefully weighed, but his persistently low sodium levels eventually led to its discontinuation. Quetiapine was increased and decreased in response to sodium trends. Sodium levels improved when medications like aripiprazole were adjusted and when supportive measures such as increasing dietary salt and managing fluid intake were implemented. 

The management of hyponatremia in his case required a multifaceted approach. Initially, the treatment included adjusting his medications to mitigate their impact on sodium levels. Paliperidone was replaced with aripiprazole 10 mg daily, a medication with a more favorable renal profile and lower risk of fluid retention compared to other SGAs. Aripiprazole, a partial dopamine agonist, is less likely to cause hyponatremia, making it a preferred choice in patients with compromised renal function. Over the next two weeks, the patient’s sodium levels improved slightly, rising to 128 mmol/L. However, the improvement in sodium levels was transient, and his psychiatric symptoms remained unstable, with recurrent episodes of agitation and paranoia. The interdisciplinary team, consisting of psychiatrists and nephrologists, carefully monitored his sodium levels while adjusting his psychiatric treatment.

Given the patient’s continued psychiatric instability, quetiapine 50 mg at bedtime was introduced to address mood lability and reduce irritability. Over the next several months, quetiapine was gradually increased to 150 mg once daily as his psychiatric symptoms fluctuated, requiring regular adjustments to his treatment regimen. Despite these interventions, his sodium levels remained unstable, fluctuating between 120 and 130 mmol/L. Adjustments in medication were made based on sodium levels. The complexity of his condition is partly due to the interplay between his CKD and the side effects of psychotropic medications. His renal impairment means that his kidneys are less capable of handling sodium and fluid shifts, which can lead to imbalances even with minor changes in his treatment or diet.

Recognizing the complexity of the case, the team implemented dietary modifications to support sodium balance. These included increased salt intake and fluid restriction, aimed at mitigating the effects of SIADH and preventing further drops in sodium levels. Care was taken to avoid overly rapid correction of sodium, which could lead to osmotic demyelination syndrome, a potentially life-threatening complication. Close monitoring of sodium levels was maintained throughout, with regular serum sodium checks.

By the end of 2023, after ongoing medication adjustments and dietary interventions, the patient’s sodium levels showed signs of stabilization, with serum sodium consistently around 130 mmol/L. While this represented a partial improvement, further optimization of his treatment was necessary. In late 2024, his sodium levels finally reached 135 mmol/L, marking a significant turning point in the management of his hyponatremia. This improvement coincided with better psychiatric control, as the patient exhibited fewer psychotic relapses and his mood remained more stable.

At this stage, his treatment regimen included aripiprazole 10 mg once daily, quetiapine 150 mg at bedtime, mirtazapine 30 mg once daily for depressive symptoms, and clonazepam 0.5 mg once daily for anxiety. Sodium levels continued to be closely monitored, particularly given the patient’s history of CKD and recurrent hyponatremia, which required ongoing adjustments to both his psychiatric medications and his diet.

This case highlights the challenges of managing antipsychotic-induced hyponatremia in a patient with complex comorbidities, including CKD. The need to balance psychiatric stabilization with sodium correction requires a personalized approach involving close interdisciplinary collaboration and frequent medication adjustments. The patient’s renal impairment limited the management options for fluid and sodium regulation, making the cautious use of antipsychotic medications like aripiprazole, known for its lower risk of inducing SIADH, crucial in stabilizing both psychiatric symptoms and sodium levels. Successful management relied on continuous monitoring of psychiatric status and electrolyte balance, ensuring that both areas were addressed without compromising the other. Moving forward, regular sodium checks and psychiatric assessments will be essential to maintaining long-term stability and preventing future episodes of hyponatremia.

## Discussion

The cases presented in this series underscore the clinical challenges associated with managing antipsychotic-induced hyponatremia. In all three cases, patients experienced fluctuating sodium levels, necessitating complex adjustments to their psychiatric treatment regimens. The balance between maintaining psychiatric stability and correcting hyponatremia was particularly difficult to achieve, as discontinuing or reducing antipsychotic medication frequently led to a relapse in psychotic symptoms.

In Case 1, the patient’s sodium levels declined rapidly after the initiation of olanzapine and long-acting haloperidol, ultimately requiring intravenous sodium chloride and dietary adjustments to manage the electrolyte imbalance. Despite these efforts, the patient’s sodium levels continued to fluctuate until sodium valproate was discontinued and lithium was introduced. Lithium’s ability to stabilize sodium levels while providing mood stabilization suggests it may be a valuable option in managing similar cases, though careful monitoring is required.

In Case 2, recurrent mild hyponatremia persisted despite the patient’s relatively stable psychiatric condition. Haloperidol, olanzapine, and sodium valproate were identified as contributing factors, and their gradual discontinuation, alongside the introduction of lithium, resulted in partial improvement of sodium levels. However, the need for continued psychiatric medication complicated the management of hyponatremia, emphasizing the difficulty in achieving both psychiatric and medical stability in such cases.

Case 3 presented a more complex scenario, with the patient’s CKD playing a significant role in his recurrent hyponatremia. Paliperidone long-acting injection, initially used to manage his psychiatric symptoms, likely contributed to the persistent electrolyte imbalance. The decision to switch to aripiprazole, a medication with a lower risk of inducing hyponatremia, resulted in a modest improvement in sodium levels. However, the patient’s psychiatric symptoms remained difficult to manage, and his treatment required ongoing adjustments to balance the competing demands of psychiatric stabilization and electrolyte correction.

The relationship between antipsychotic use and the development of hyponatremia is complex and multifaceted. Numerous studies have indicated a significant association between these medications and the onset of hyponatremia, particularly through mechanisms that involve the inappropriate secretion or action of ADH, leading to conditions such as SIADH or NSIAD. The literature suggests that antipsychotics, particularly SGAs like risperidone and olanzapine, can stimulate V2R in the kidneys, leading to increased water reabsorption. This process is often independent of elevated ADH levels and results in dilutional hyponatremia [1,2].

Clinical evidence from large-scale studies, such as the Swedish population-based case-control study, supports the correlation between antipsychotic use and hospitalization for hyponatremia, with first-generation antipsychotics (FGAs) showing a particularly strong association. The mechanisms underlying this relationship are believed to involve both direct pharmacological effects on the kidneys and indirect effects mediated through the central nervous system’s regulation of water and sodium balance. FGAs are thought to exert a more pronounced effect on these pathways, which might explain their stronger association with severe hyponatremia compared to SGAs [3].

However, the evidence is not entirely consistent across all studies. For example, another study, by Jessani et al., found no significant correlation between antipsychotic use and sodium levels in patients with chronic schizophrenia, suggesting that other factors might contribute more significantly to the development of hyponatremia in certain patient populations. This discrepancy highlights the need for further research to clarify the conditions under which antipsychotics are more likely to induce hyponatremia and to identify which patient populations are at the greatest risk [4].

The management of antipsychotic-induced hyponatremia poses considerable challenges for clinicians, as this condition can lead to severe complications, including neurological symptoms such as confusion, seizures, and, in extreme cases, coma. The risks are especially pronounced in vulnerable populations, such as the elderly, who are more susceptible to falls and fractures due to the cognitive and motor impairments caused by hyponatremia. Even mild hyponatremia in these individuals can significantly elevate the risk of adverse outcomes, requiring timely and effective intervention [5].

One of the primary dilemmas in treating antipsychotic-induced hyponatremia is balancing the need to maintain psychiatric stability with the risk of exacerbating electrolyte imbalance. Discontinuation of the offending antipsychotic is often necessary to correct the hyponatremia, but this approach risks triggering a relapse of psychiatric symptoms, which can be destabilizing for the patient. In cases where discontinuation is not feasible, dose reduction or switching to an alternative antipsychotic with a lower risk of inducing hyponatremia, such as aripiprazole, may be considered. However, these interventions must be carefully managed to avoid exacerbating psychiatric instability or relapse [6].

Fluid restriction is a commonly recommended first-line treatment for SIADH-related hyponatremia, but its effectiveness can be limited, particularly in patients who are unable or unwilling to comply with strict fluid intake limits. In more severe cases, the use of vasopressin receptor antagonists, such as tolvaptan, or hypertonic saline may be necessary to rapidly correct sodium levels. However, these treatments carry their own risks, including the potential for overly rapid correction of sodium levels, which can lead to osmotic demyelination syndrome, a serious and potentially fatal condition [3,5].

The literature also emphasizes the importance of regular monitoring of sodium levels, particularly during the early stages of antipsychotic therapy or when doses are increased. Baseline sodium levels should be assessed before initiating treatment, and patients should be closely monitored for any signs of hyponatremia, especially if they belong to high-risk groups, such as the elderly or those with a history of electrolyte imbalances [4,5]. Early detection and intervention are crucial in preventing the progression of hyponatremia to more severe stages, where the risks of complications and treatment difficulties are significantly higher [5].

Despite the challenges in managing antipsychotic-induced hyponatremia, it is important to recognize that not all patients treated with antipsychotics will develop this condition. The variability in risk among different antipsychotics and patient populations suggests that individualized treatment plans are essential. Clinicians must weigh the benefits of antipsychotic therapy against the risks of hyponatremia, taking into account the specific needs and vulnerabilities of each patient [1,4].

In conclusion, while the correlation between antipsychotic use and the development of hyponatremia is well-supported by the literature, there remains a need for further research to better understand the mechanisms involved and to develop more effective strategies for managing this condition. The cases presented in this report highlight the complexities and challenges of treating hyponatremia in patients on antipsychotic therapy and underscore the importance of a careful, individualized approach to treatment that considers both the psychiatric and medical needs of the patient. By continuing to explore the relationship between antipsychotics and hyponatremia, clinicians can improve patient outcomes and reduce the risks associated with this serious electrolyte disturbance [1,3,5].

## Conclusions

This case series highlights the significant clinical challenge of managing hyponatremia induced by antipsychotic medications, particularly in patients with comorbidities such as CKD. The persistent fluctuation of sodium levels and the associated risks required frequent and meticulous monitoring. The treatment of these patients was further complicated by the need to balance psychiatric stabilization with the correction of sodium imbalances. Adjustments in psychotropic medication regimens, such as the introduction of lithium, contributed to both mood stabilization and improved sodium balance. Additionally, aripiprazole played a pivotal role in reducing the risk of further hyponatremia while maintaining psychiatric stability too. The discontinuation of medications known to exacerbate hyponatremia was essential in restoring more stable sodium levels and improving overall patient outcomes. The cases demonstrate the complex interplay between antipsychotic therapy and electrolyte disturbances, emphasizing the need for a personalized, interdisciplinary approach. Close monitoring of both psychiatric symptoms and serum sodium levels is crucial to ensure successful long-term management and to prevent further episodes of hyponatremia.
